# 

**Published:** 1868-01

**Authors:** 


### BIBLIOGRAPHICAL NOTICES.

*Chemical Essays in Reference to Dental Surgery.*—By Prof. George Watt, M.D., D.D.S., of the Ohio Dental College. Published by S. S. White. We are glad to have these interesting *Register Papers* in the form of a handsome little volume of two hundred and sixty pages, and recommend the work to all our readers. A perusal of it will convince every one that it is a valuable addition to the literature of our profession, and we can only regret that Prof. Watt has not seen fit to treat at greater length, some of the subjects upon which these interesting essays have been written.

The neat appearance of the work does credit to the publisher.

*The Teeth: Their Health, Disease and Treatment.*—By J. P. H. Brown, Dentist, Augusta, Georgia. The author announces in the preface that this little work has been written to instruct the people, and that he has divested his description, as far as possible of technical language. That he has succeeded in his efforts to present the subjects treated of in an intelligible manner, is apparent on a perusal of the work, which, though small, contains much useful information.

*Transactions of the American Dental Association*—This is a full account of the proceedings of this Association at the meeting held in Cincinnati, July 30th to August 3d inclusive, 1867, in a volume of one hundred and thirty-three pages, which contains much useful information.

